# Understanding University Students’ Experiences of Engaging With AI and Apps for Their Mental Health and Well-Being: Qualitative Study

**DOI:** 10.2196/75381

**Published:** 2026-06-30

**Authors:** Julia Groot, Ben Ainsworth, Nathalia Gjersoe, Christopher Clarke, Richard Hamshaw, Mark Brosnan

**Affiliations:** 1 Department of Psychology, University of Bath, Claverton Down, Bath, BA2 7AY, United Kingdom, 44 01225388388; 2 School of Psychology, Faculty of Environmental and Life Sciences, University of Southampton, Southampton, United Kingdom; 3 Department of Computer Science, University of Bath, Bath, United Kingdom; 4 Centre for Applied Autism Research, Department of Psychology, University of Bath, Bath, United Kingdom

**Keywords:** AI, apps, mental health apps, MHapps, mental health, well-being, support, promotion, engagement, artificial intelligence

## Abstract

**Background:**

Fully automated digital technologies, such as artificial intelligence (AI) and apps, provide a particularly promising way to promote and support mental health and well-being in university students due to their accessibility, scalability, and cost-effectiveness, among other factors. Nevertheless, they are currently impeded by suboptimal engagement and high dropout rates, limiting their effectiveness to promote and support mental health and well-being.

**Objective:**

This study aimed to understand university students’ experiences of engaging with AI and apps to promote and support mental health and well-being.

**Methods:**

University students who did not currently experience a mental health condition were recruited to ensure a nonclinical sample. Qualitative semistructured interviews were adopted and focused on students’ experiences of engagement with AI and apps for their mental health and well-being. These interviews were conducted in April and May 2023 and lasted 30 minutes, 2 seconds (SD 9 min, 49 s), on average. Interviews were transcribed verbatim and analyzed using a reflexive thematic analysis.

**Results:**

A total of 21 interviews were conducted, and 4 main themes and 4 subthemes were constructed. The first main theme refers to the “need” to engage with AI and apps for mental health and well-being. Specifically, this theme describes how nonclinical students would primarily use these technologies as a support strategy when their mental health and well-being deteriorate, and their preexisting mental health and well-being strategies are insufficient. The second theme refers to AI and apps as both a barrier and solution to stigma; while students are less inclined to access mental health apps due to stigma, they also consider apps to be less intrusive compared with other forms of support. The third theme considers a lack of trust in AI and apps. This lack of trust primarily exists due to skepticism about the capabilities of AI and apps supporting and promoting mental health and well-being, and skepticism about their ability to safeguard mental health and well-being. The final theme describes how usage is dependent on unique AI and app characteristics. Students may engage more in AI and apps when humanity, warmth, and care are considered less crucial, and when a lack of judgment and pressure is considered imperative.

**Conclusions:**

Overall, nonclinical university students were more likely to engage with AI and apps when they experienced a decline in their mental health and well-being. Thus, it could be more beneficial to adopt apps as a support strategy rather than as a promotional strategy in a nonclinical sample. Furthermore, future policy and practice should implement strategies to safeguard mental health and well-being and provide open and honest communication about the capabilities of AI and apps in order to build trust and enhance engagement with digital technologies for mental health support.

## Introduction

### Background

Mental well-being is a complex construct that is commonly defined to include 2 perspectives—the hedonic and the eudaimonic perspective [1]. The hedonic perspective refers to a subjective experience (often summarized as happiness), which includes satisfaction with life, the presence of a positive mood, and the absence of a negative mood [2]. The eudaimonic perspective is defined as a process of self-realization that contains six dimensions, including (1) autonomy, (2) purpose in life, (3) personal growth, (4) positive relations with others, (5) environmental mastery, and (6) self-acceptance [3]. Mental well-being can be promoted through activities that aim to enhance, build, or strengthen individuals’ strengths, resourcefulness, or resiliency [4]. The main aim of mental well-being promotion is to enhance or maintain good levels of mental well-being. However, mental well-being and mental health are closely related, and therefore, mental well-being promotion often simultaneously improves mental health outcomes (such as anxiety and depression) [5].

A large proportion of nonclinical university students experience suboptimal levels of mental well-being [6-8]. This is likely due to the significant lifestyle changes university students experience, such as moving away from home, academic and financial pressures, and a loss of social and emotional support networks [9]. Additionally, research has suggested that approximately 25% of university students experience depression and anxiety [10], and this prevalence of mental health conditions in university students continues to rise. While many universities identify a duty of care for students’ mental health and well-being, support services at universities are often overstretched [9], thereby limiting students’ access to mental health and well-being support. This highlights the importance of accessible care in a nonclinical student population to prevent the onset of mental health conditions and improve their mental well-being.

A particularly promising way to deliver accessible mental health and well-being support to nonclinical university students is through fully automated digital technologies—digital technologies that do not require any form of ongoing human input to be delivered [11]. Automated digital technologies include apps that can be accessed through technological devices, such as smartphones and tablets [11]. Apps are designed to perform specific tasks, such as the delivery of activities for mental health and well-being. However, apps can also include generative artificial intelligence (GenAI), which allows for greater personalization of content, or they can rely entirely upon artificial intelligence (AI; eg, large language models) [12]. The automated delivery through apps and AI allows for mental health and well-being support to be highly scalable and cost-effective, thereby providing an easily accessible way to access support [13]. Unsurprisingly, mental health and well-being apps are becoming increasingly prevalent. The apps market size of US $6.25 billion in 2023 is expected to continue growing at 15.2% annually, with young adults (including university students) being its primary user [7,14].

A recent systematic review and meta-analysis indicates that automated apps and AI can effectively improve mental well-being in the nonclinical population [15]. Systematic reviews and meta-analyses also indicate that apps can effectively reduce mental health symptoms alongside improving mental well-being [16]. Additionally, AI has been shown to effectively support mental health [17]. Despite this great potential, studies have shown that these technologies suffer from suboptimal levels of engagement and high levels of dropout, thereby not reaching their full potential [15,18].

Some research has been conducted to understand engagement with specific mental health and well-being apps. Specifically, research investigated student engagement after providing them with access to a mental health app “Thought Spot” (Thought Spot was created through participatory design research methods and usability testing with college and university students [19]) for 6 months [20]. They found important app-specific determinants of continued engagement, such as detailed and inclusive content enhancing engagement, and technical errors and a lack of integration with other apps reducing engagement. Further research aimed to understand participants’ engagement with mood-tracking apps [21]. They found further context-specific determinants of engagement, such as enjoying keeping track of trends in their moods through calendars. Similarly, research found app-specific determinants of engagement with a mental health promotion intervention, such as engaging and accessible app content [22].

Borghouts et al [23] conducted a review on barriers to engagement across different mental health and well-being apps, investigating studies from 2010 to 2019. However, this review did not adopt a nonclinical university student sample, which is particularly important since the review found mental health status to be an important determinant of engagement [23]. Furthermore, tremendous digital developments have occurred since then, and the review does not include perspectives regarding engagement with more advanced technologies, such as AI. Melcher et al [24] further conducted an empirical study to understand engagement across different mental health apps in college students, but did not conduct an in-depth qualitative analysis and may therefore provide less depth toward the contextual understanding of engagement [25]. Jardine et al [26] conducted an exploratory analysis of questionnaire and interview responses regarding engagement with mental health apps. However, this study focused on nonengagers. Overall, there is a need for research into engagement with AI and apps for mental health and well-being in a nonclinical student population, adopting in-depth qualitative analyses.

### Research Aim

Therefore, this study aimed to understand university students’ experiences of engaging with AI and apps for their mental health and well-being.

## Methods

### Study Design

This study adopted semistructured qualitative interviews.

### Epistemological Position

A constructivist epistemological stance was taken in this study. This approach was adopted as it identifies that the researcher, interviewing and analyzing the data, plays a role in the construction of truth and meaning [27]. The approach identifies that people can construct the truth or meaning differently in similar situations. Therefore, it was considered important to reflect on the researchers’ subjectivity and its impact on the data and findings by adopting a reflexive thematic analysis (TA) [28].

### Participants and Recruitment

Participants were recruited via convenience sampling at the researchers’ institution. A study advert (with relevant information regarding the study and a link to sign up) was promoted at sites at the researchers’ institution, and via online university channels. University channels used to advertise the study targeted students in the Department of Psychology and Computer Science.

To ensure the confidentiality of participants, no demographic information was collected. Particularly, since demographic characteristics (such as age, gender, year of study, and department) in combination with qualitative interview data were considered to lead to highly identifiable data. Therefore, the general demographics of students in both departments were obtained instead.

### Sample Size

The sample size was considered using the information power principle. This suggests that the more information a sample holds, the smaller the sample is needed [29]. This study adopted a relatively broad study aim and cross-case analysis, but a dense sample specificity, use of an established theory, and strong dialogue. Therefore, the initial aim was to recruit 15‐20 participants.

### Eligibility Criteria

A convenience student sample was recruited. Eligible participants for this study were (1) university students, (2) aged more than 18 years. Participants were excluded if they (1) were receiving treatment or psychopharmacological drugs for a mental health condition at the time of the study, (2) they scored ≥10 on the Patient Health Questionnaire-8, and (3) they scored ≥8 on the Generalized Anxiety Disorder-7. This was to ensure participants’ mental health could be considered “nonclinical,” as fully automated digital technologies are considered particularly beneficial as universal interventions targeted at the general nonclinical population [30].

There were no specific inclusion or exclusion criteria regarding students’ experiences with mental health and well-being apps. Therefore, some students were currently using or had used several mental health and well-being apps, while other students had never used them before. Furthermore, the apps that students had used included a range of different ones, and the results are therefore not specific to an app. This was to ensure general perspectives and experiences regarding engagement across mental health and well-being apps were considered. Nevertheless, inductive coding was adopted to identify the mental health and well-being apps (including AI) that students referred to during the interviews.

### Materials

A semistructured interview schedule was developed for the purpose of this study (Part S1 in Multimedia Appendix 1). This interview schedule adopted several open-ended questions aimed at understanding students’ experiences of using AI and apps for their mental health and well-being. Since the initial focus of the interview was entirely on mental well-being promotion, participants were asked about their understanding of “mental well-being promotion” and provided a definition by the researcher (refer to Introduction section) to ensure participants had a consistent awareness of the meaning of this construct.

### Procedure

Students expressed their interest in participating in the study by signing up via an online questionnaire link on the study adverts. This link provided participants with the information sheet (Multimedia Appendix 2), after which they were taken to the screening questionnaires (Patient Health Questionnaire-8 and Generalized Anxiety Disorder-7). After filling out the screening questionnaires, participants who were not eligible were provided with relevant information and resources for their mental health and well-being. Participants who were eligible were contacted by the researcher (JG) via email to schedule a convenient date and time for the interview to take place. The interviews took place in the laboratories at the researchers’ institution between April 19 and May 31, 2023. Upon arrival in the laboratory, informed consent was obtained (Multimedia Appendix 3). Following this, a brief explanation was provided, and the interview was conducted. The interview consisted of 2 separate parts, lasting a maximum of 1 hour in total. The first part included a semistructured interview regarding participants’ views of apps and AI for mental health and well-being support; this part of the interview was analyzed and is presented in this study. The second part of the interview involved participants looking at app content and sharing their thoughts out loud; this part of the interview was analyzed in a separate study. Refer to Multimedia Appendix 1 for the interview schedule, including both parts of the interview. Once the interviews finished, participants were provided with a debrief form (Multimedia Appendix 4) and student credits or a £10 (US $13.52) gift voucher to thank them for their time.

### Ethical Considerations

Ethical approval was obtained from the Psychology Research Ethics Committee at the University of Bath (reference 23 042). Participants were first presented with an information sheet when they accessed the online expression of interest questionnaire. They were presented with the information sheet again at the start of their interview in the laboratory. Participants were provided with an opportunity to ask questions, and informed consent was obtained in writing before data collection. Participants had the opportunity to withdraw during the interview by informing the interviewer. After the interview was completed, participants could withdraw their data until May 31, 2023. Participants were informed about this on the information sheet. To ensure confidentiality, any identifiable information was anonymized during transcription, and participants were assigned pseudonyms, which are used in this publication. Furthermore, to mitigate participant burden, combined duration of Part 1 and Part 2 of the interview (with Part 2 reported elsewhere) remained within a 1-hour time limit. Aftercare was provided if necessary. Specifically, if sensitive topics were discussed and/or concerns regarding mental health and well-being were raised during the interview, a discussion between the researcher and the supervisory team was conducted to identify the most appropriate form of aftercare—such as the university well-being apps and/or other mental health support services.

### Data Analysis

Data was analyzed using a reflexive TA [28]. This was considered the most appropriate method of analysis to identify different themes and patterns of meaning regarding students’ engagement with AI and apps for their mental health and well-being. An inductive approach was adopted to analyze the data. The interviews were audio-recorded using an Olympus VN-541 PC high-quality digital voice recorder. The part of the semistructured interviews reported in this study lasted 30 minutes 2 seconds (SD 9 min 49 s) on average, ranging from 16 minutes 20 seconds to 49 minutes 37 seconds. The recordings were transcribed using an orthographic (verbatim) transcription method. This method was adopted as it was decided that only information relevant to understanding and conceptualizing the meaning would be necessary to answer the research question and would therefore be transcribed. Interview transcripts were fully anonymized, and each participant was assigned a pseudonym.

The six phases of TA proposed by Braun and Clarke [28] were used to analyze the primary research question: (1) familiarization with the data took place, (2) transcripts were coded, (3) themes were generated, (4) themes were reviewed, (5) final defining and naming of themes, and (6) write-up took place. Specifically, a reflexive journal was kept throughout the analytical process, and continuing discussions took place between author JG and the research team to further conceptualize meaning. The transcripts were coded by author JG using NVivo 14 (Lumivero), adopting an inductive interpretive approach, which at times was more descriptive in nature. Codes were regularly revisited by JG to conceptualize and construct meaning. Initial themes were constructed by JG, which were further developed and reviewed in discussion with coauthors.

### Reflexivity

#### Research Team

This study was undertaken by an interdisciplinary research team. The research team consisted of a PhD student in digital mental health and well-being interventions (JG), an associate professor in digital health and behavior change (BA), a professor in cognitive developmental psychology and an associate pro-vice-chancellor in student experiences (NG), a lecturer in human-computer interactions (CC), a senior lecturer in social psychology and qualitative research methods (RH), and a professor in autism spectrum disorder adopting digital technologies (MB).

#### Positionality

Author JG adopts a position as both an outsider (researcher) and an insider (peer or student) in this study. The remaining research team could be considered outsider researchers. Adopting a position as an insider researcher inevitably creates preconceptions regarding students’ experiences of engaging with apps for mental health and well-being, shaping the construction of themes in this study. Furthermore, research found that important factors for self-disclosure include the recipient being considered trustworthy and confidential—qualities which may have been more likely to be instilled in an (outsider) researcher [31]. Therefore, it was prioritized to instill trust through the study environment (university laboratories), the interviewer (JG) introducing herself as an (outsider) researcher, and highlighting data confidentiality at the start of the interview.

## Results

### Overview

A total of 21 participants took part in this study. The initial estimation of sample size (guided by information power) was continually appraised during the analytic process [29]. Specifically, the authors identified that participants frequently conflated mental well-being promotion and mental health support during interviews. While there is considerable overlap between mental well-being promotion and mental health support, important differences exist. For example, mental well-being promotion primarily aims to maintain or improve mental well-being outcomes and is often delivered through universal interventions targeting the entire nonclinical population, while mental health support primarily aims to support mental health (such as anxiety and depression) and is often delivered to targeted or at-risk populations [30]. Despite this, conflation highlighted the need to revise the initial study aim to include mental health support alongside mental well-being promotion. This revision expanded the scope of the study aim, and therefore, it was agreed that additional participants were required to capture the thematic complexity. The final sample size was then determined using code saturation [32]. Author JG identified code saturation (whereby no new codes were identified) after 21 interviews. Code saturation was then verified through discussion with the research team.

Demographic characteristics of the population from which participants were recruited are presented in Table 1.

**Table 1.** Demographic characteristics for the 2022-2023 student cohort in the departments of Psychology and Computer Science.

Characteristics	Department of Psychology (N=1120), n (%)	Department of Computer Science (N=1725), n (%)
Sex		
Male	171 (15.25)	1353 (78.43)
Female	949 (84.75)	372 (21.57)
Age (years)		
18-20	777 (69.33)	640 (37.1)
21-24	199 (17.78)	295 (17.1)
25+	144 (12.89)	790 (45.8)
Level of education		
Undergraduate	780 (69.64)	645 (37.39)
Postgraduate taught	200 (17.86)	995 (57.68)
Research	140 (12.5)	85 (4.93)
Total number of students	1120 (100)	1725 (100)

Inductive coding identified a range of mental health and well-being apps and AI technologies referred to by participants during the interviews. These include habit and mood trackers, journaling, exercise, sleep, mindfulness, productivity, university well-being apps, mental health support via rule-based and AI-based chatbots, as well as AI therapists, AI to tailor or personalize content within apps, and general large language models, such as ChatGPT (OpenAI). For a full overview of the apps and AI participants (explicitly) referred to during interviews, refer to Multimedia Appendix 5. Some of the below findings referencing these apps and AI tools for mental well-being promotion and mental health support are discussed hypothetically, while others may reflect participants’ actual experiences.

### Summary of Themes and Subthemes

A total of 4 main themes and 4 subthemes were constructed by the authors to provide an understanding of students’ experiences of engaging with AI and apps to promote and support their mental health and well-being (Table 2).

**Table 2.** Overview of constructed themes and subthemes.

Themes and subthemes	Characteristics
Experiencing a “need” to engage	This theme refers to the importance of having a mental health and well-being “need” to engage with AI^a^ and apps. This “need” primarily occurs when preexisting mental health and well-being strategies are considered insufficient. Therefore, AI-based apps and apps are often not considered as “promotional tools” but instead as “support” for mental health and well-being.
A barrier and solution to stigma	This theme identifies stigma as a barrier to engagement with AI and apps for mental health and well-being support, potentially due to these tools primarily being considered as a “support” rather than a “promotion” strategy. It further identifies that AI and apps could potentially help reduce this stigma and “normalize” accessing support through stepped care.
Lack of trust	
Insufficient capability to support and promote mental health and well-being?	This subtheme refers to the skepticism students hold which works as a barrier toward engagement with AI and apps to promote their mental health and well-being. Specifically, it refers to the skepticism students have about these technologies’ current and general ability to promote and support mental health and well-being.
An inability to safeguard mental health and well-being?	This subtheme refers to students’ skepticism toward AI and apps due to the perceived inability to, and prioritization of, safeguarding of mental health and well-being in AI and apps, thereby reducing their engagement with apps.
Usage dependent on unique AI and app characteristics	
No judgment and pressure	This subtheme highlights how the unique AI and app characteristic “no judgment and pressure” facilitates engagement in certain situations. It identifies how an inability to judge and less perceived pressure in AI and apps provides nonintrusive access to mental health and well-being support.
A lack of humanity, warmth, and care	This subtheme highlights situations in which individuals may be more and less likely to engage with AI and apps depending upon the unique characteristic “lack of humanity, warmth, and care.” It highlights that although humanity, warmth, and care are lacking, this could be considered a barrier as well as a facilitator to engagement, depending on each specific situation.

a AI: artificial intelligence.

### Theme 1: Experiencing a “Need” to Engage

Most students identified that they did not engage with AI and apps for their mental health and well-being, as they did not currently experience a mental health or well-being “need.” Students mentioned that this lack of “need” was due to their mental health and well-being being “good” at that moment.

*I don’t feel the need to, I guess … Just because I think that, uhm, I do think that I could gain sort of a greater sense of well-being from it, but … I have sort of a general quite high level of well-being. And so although I think that it could promote my well-being further, it’s not the most sort of pressing concern for me currently.*

[Jack]

Jack’s account was interpreted as positioning engagement with AI and apps as conditional on experiencing lower well-being; since he currently feels his well-being is “quite high,” Jack does not perceive a “need” to engage at this time. Interestingly, this contradicts theory that considers self-actualization a “growth need”—a need that occurs out of a desire to grow rather than a deficiency [33]. However, perhaps students perceive mental health and well-being as a deficiency need instead of a growth need. Deficiency needs motivate people when they are unmet, and once a deficiency need has been satisfied, it goes away [34]. Indeed, students identified that they used AI and apps in times of “need,” but discontinued their use once this “need” was satisfied and went away.

*If I feel like … after the first session or few sessions I feel like I’ve- already getting better like, like a bit improving, and I’ll probably like stop using it for a bit, I guess like oh, maybe I don’t need any more because I'm like, become I guess more stable enough [Interviewer: “mm-hmm”] to stop using it until I need it next time.*

[Brenda]

Brenda identified that once the “need” to engage disappeared as her mental health improved, she would discontinue using the intervention. This may suggest students are not motivated to self-actualize through AI and apps and perceive these as relevant tools to satisfy a deficiency need instead.

Nevertheless, it may be consistent with the technology acceptance model (TAM). This model suggests that engagement with AI and apps occurs when this is considered “useful” [35]. Perhaps engaging when there is no mental health “need” might be considered less useful. Instead, most students mentioned that they would rather engage in other activities to promote their mental well-being and self-actualize.

*If I want to promote my mental well-being, you know, I’ll just go for a run after my lecture or something.*

[Elsa]

*[Playing squash] is quite good to like switch off and it’s quite a social thing as well. … I’m enjoying it.*

[Holly]

Holly and Elsa identified that when there is no immediate “need” to work on their mental health and well-being, they prefer engaging in other (nondigital) activities for their mental health and well-being. This might be because most students identified that they experience more joy and further benefits, such as connectedness and improvements to their physical health, when pursuing other activities. This may suggest that automated digital activities do not lead to the same satisfaction of basic psychological needs. According to the basic psychological needs theory (BPNT), the satisfaction of (1) autonomy (volition and willingness which lead to a sense of authenticity), (2) relatedness (connection, experiencing warmth, bonding, and care), and (3) competence (the experience of mastery to use and extend skills and expertise) are essential for optimal mental health and well-being [36]. Hence, perhaps students pursue other well-being activities as they lead to greater satisfaction of basic psychological needs.

Since students had already implemented these other well-being activities in their daily lives, they would also often prefer to stick to these activities instead of implementing a novel app or AI activity in their daily lives. They mentioned that only when these activities did not provide the necessary support or effect for their mental health and well-being, would they consider adopting novel mental well-being strategies delivered through automated digital technologies.

*If there is an issue with my friend group, [Interviewer: “Mm-hmm”] and I only share my problem with my friends in a situation like that, the app would be useful where I could share it with someone who is not in that situation.*

[Molly]

Molly highlights that her friend group would be her mental health and well-being support strategy. Whenever she is unable to access this support strategy, she would consider looking at novel support strategies such as apps and AI. Molly’s account was interpreted to position apps as a secondary option—useful when her primary support system (friends) is unavailable—supporting the construction of the theme of need-dependent engagement.

Although students mention they are interested and open to using automated digital technologies for their mental health and well-being in times of “need,” they also identified that adopting these self-help strategies when in “need” could be challenging. Primarily, these interventions require individuals to self-manage their condition, rather than receiving more intensive support and guidance, such as through traditional face-to-face support methods. Adopting self-help in times of “need” could be particularly challenging when experiencing reduced mental capacity.

*If … I feel quite stressed, I feel like having to then, perform my [app activity], might feel unreachable, not realistic?*

[Henry]

Henry identifies that engaging with automated digital technologies to perform a self-guided activity could feel “unreachable” and “unrealistic” when in times of “need.” Similarly, research has found that the majority of students are interested in using an app for their mental health, but that only a small proportion of students (16/149, 10.7%) have actually engaged with apps during times of need [37]. This might indicate the challenge to engage with automated digital technologies when in times of “need.”

### Theme 2: A Barrier and Solution to Stigma

Another important factor impacting student engagement is public and self-stigma. While it has been suggested that AI and apps are associated with less stigma compared with traditional (nondigital) mental health support services [13], it is important to recognize that stigma still plays an important role, thereby reducing engagement. Several students identified that accessing any psychological intervention—including ones delivered digitally—is stigmatized. Perhaps this is unsurprising as stigma is considered a prominent barrier to help-seeking generally [38].

***Interviewer:** Is there anything that stops you, uhm, at the moment from using a mental well-being promotion app?*

***Jack:** Probably the stigma of using it. Uhm, as I go back to, it doesn’t seem normalized, I don’t know anyone who uses it.*

Jack highlights that stigma, mental health and well-being apps, and AI not being normalized, are important reasons for him not to engage. This is in line with research, which has found that self-stigma (someone’s internalized attitudes) [39] has been linked to reduced help-seeking behaviors [40,41]. Jack shows self-stigma by mentioning that he personally does not think it is normalized, as he does not see anyone using it. Nevertheless, this contradicts recent findings that mental health and well-being apps are downloaded over a million times monthly [42].

However, stigma is understood as more prominent when apps have been developed for specific mental health purposes (such as an AI-based therapist) as opposed to low-key well-being activities (such as journaling and mindfulness meditation). It was also understood as a more prominent barrier when digital technologies are developed by recognized mental health organizations (such as universities, government, and mental health charities) compared with the private sector.

*Uhm, there might be a s-s-stigma? Like using the university one [university mental health app]? Opposed to like using … like headspace, and stuff like that.*

[Elsa]

*Depends a little bit as well on what exactly it is that you’re doing in that app. [in overlap] If it’s- if it’s for example, something like journaling, and there might be less stigma to than more of an AI focused kind of therapist.*

[Jack]

Elsa and Jack suggest that the barrier “stigma” to engage with AI and apps for mental health and well-being is perhaps not uniform, suggesting that students perceive greater stigma when tools appear more clinical or institutionally linked. This likely arises from the perception that accessing more intensive psychological support, such as AI and apps developed by recognized mental health organizations, is associated with weakness. Yap et al [43] refer to this specific kind of stigma as “weak-not-sick beliefs,” which is further associated with lower intentions to seek support. Indeed, several students mentioned that admitting to a decline in their mental health and well-being to an extent where they “need” additional support is stigmatized. Therefore, students adopt other strategies before admitting to this “need” for support and accessing AI and apps.

*I’ll like do an exercise or something like, uhm, like try to like calm down before I use any of these apps, because I feel like they’re still a bit of … a bit of a pressure for me to use these apps. … I feel like I try to stay away from like, a professional kind of help, like, I’m quite an independent person.*

[Harry]

Harry mentions he would try another activity first, as he tries to stay away from “professional help.” Harry’s emphasis on independence demonstrates how internalized stigmatizing beliefs about help-seeking shape reluctance to engage with digital tools. Harry mentions that he is an “independent person,” which indicates that he perceives individuals who access support as not being independent and could relate to the “weak-not-sick belief.” This again highlights the stigma associated with accessing mental health support generally, and therefore, apps for mental health and well-being support too. Nevertheless, mental well-being promotion might also suffer from this barrier, as promotion and support were often considered interchangeably by students.

Additionally, stigma was further shown in students hiding that they were accessing support. Students identified that it is easier to hide that they are accessing support when accessing AI and apps, but even then, they expressed concerns that someone else could find out.

*So if, for example, someone has your phone and sees that, “oh, that person has a mental health app.” That could potentially make someone less likely to download that app because they will think “oh, what if people think that there’s something wrong with me?”*

[Ralph]

Ralph mentions that someone finding a mental health and well-being app on their phone could be considered a barrier to engagement. This highlights the perceived public stigma associated with accessing any psychological intervention or support, and therefore, not wanting others to be aware of accessing this support. However, this could increase engagement relative to traditional face-to-face support methods, as it may be easier to hide that you are accessing support when accessing AI and apps.

Although students highlighted stigma would prevent them from engaging with apps and AI generally, students also identified that they could potentially provide a solution to public and self-stigma.

*I think sometimes, with things that, with things like this that have a stigma. I think it takes people doing the small things first, to realise: “you know what? This isn’t so bad.”*

[Anne]

*I think taking that first step of trying something self-guided, might make people more willing to then contact them, use the messaging and contact someone in-, with the app.*

[Barbara]

Anne and Barbara highlight the importance of engaging in something low-key for mental health and well-being first, which may act as a solution to gradually reduce stigma to engage with more intensive mental health and well-being support. Indeed, Ho et al [44] suggest that a stepped-care model could reduce stigma and improve access to those in “need.” They suggest a stepped-care model in which different levels of care are provided, from less intensive care (such as self-guided interventions) to more intensive care (such as specialist treatment). Research further identifies several approaches that can effectively reduce stigma by adopting apps. For example, education-based approaches are one of the most commonly used approaches to combat stigma [45]; furthermore, mental well-being activities (such as the positive psychology activity “expressive writing”) have been found to reduce self-stigma [46]. Both of these activities could be delivered through digital technologies and thereby help reduce stigma. Thus, AI and apps could potentially provide a crucial way of reducing stigma to alter public and self-stigma and enhance engagement with mental health and well-being support accordingly.

### Theme 3: Lack of Trust

#### Overview

Many students recognized a great potential for AI and apps to promote and support their mental health and well-being. However, they currently do not engage with AI and apps due to a lack of trust. Trust is defined by Mayer et al [47] as: “a willingness of a party to be vulnerable to the action of another party based on the expectation that the party will perform a particular action important to the trustor, irrespective of the ability to monitor or control that other party.” Indeed, relevant theory suggests that trust is an important factor determining engagement with digital technologies (TAM) [35,48]. Two subthemes have been constructed, identifying prominent reasons students currently lack trust, which prevents them from engaging with AI and apps.

#### Insufficient Capability to Support and Promote Mental Health and Well-Being?

Students are aware of technologies (particularly AI) developing at an exponential rate, and expressed concerns about whether these rapidly developing technological tools are sufficiently capable to provide support for their mental health and well-being. They identified that, at times, AI can make errors and is unable to tailor to an individual’s needs.

*I seem to get really frustrated [chuckles] when it’s like a robot or like erm an AI on the other side because … there are certain things that they don’t really understand.*

[Harry]

Harry mentions that he gets very frustrated with AI when it does not understand him. Chhabra et al [49] indeed identified that a lack of semantic understanding (an inability to understand the queries), personalization when responding to the query, and competency (inability to provide a fitting solution) are some of the most common limitations in AI’s capabilities that can lead to frustration in individuals. Similarly, Molly mentions she is skeptical of apps’ abilities to support individuals’ needs.

*I would say in-person, in-person would more likely to be more focused on what the person needs because they’re probably trained [Interviewer: “mm-hmm”] so they know what to do exactly and in what conditions, which the app might not be able to do that.*

[Molly]

Harry and Molly’s views illustrate students’ doubts about whether digital tools can offer personalized, context-sensitive support. Nevertheless, their skeptical views on AI and apps’ capability to support and promote mental health and well-being may be justified. Recent research by De Freitas et al [50] indeed suggests that AI returns messages to mental health queries that are considered unhelpful in as many as 24.5% (265/1080) of cases, and messages are even considered to be risky in 38.1% (411/1080) of cases. Furthermore, they found that in high-risk situations, such as suicide and self-harm, AI returned unhelpful and risky messages, such as by suggesting talking to people with the same interests. Its capability was found to depend on the clarity of communication by the user. For example, clear, explicit queries for support were more accurately supported compared with vague support queries [50].

While this refers to the limited capabilities when communicating verbally, it does not take into account nonverbal communication. Students identified the importance of nonverbal expressions in mental health and well-being support—information that AI and apps are currently unable to access—and how this might limit the capability to support mental health and well-being. Nonverbal expressions could include facial expressions, eye contact, body movement, and affect [51]. These nonverbal cues may at times more accurately represent true emotional states than verbal cues [52]. Furthermore, Burgoon et al [53] show that approximately 60%‐65% of interpersonal communication is displayed nonverbally. Thus, students are skeptical about AI and apps’ ability to support mental health and well-being when they do not have access to any nonverbal expressions currently.

*You might not have that same level of personal care? That you get from talking to a person? You know, they can sort of see how you’re like, reacting to questions in a physical way? Like, I don’t know, sometimes you might be talking and you sort of make a face when you’re thinking about something, and that might sort of indicate to a GP or a mental healthcare practitioner that, maybe you’re not getting to the bottom of what- how you’re actually feeling. Whereas if you’re sort of messaging on an app, you might not, it might not pick up on those like nuances that an in-person appointment might sort of access.*

[Henry]

*I don’t think an app is good enough for a crisis … it’s not directly seeing the person. I think that’s what app can’t mimic, it can’t see the person, and directly see what that person, how they’re feeling [Interviewer: “mm”]. And I think, if the person’s in crisis, I just don’t think the app would b-, I don’t think it would be good enough?*

[Meghan]

While Henry and Meghan may be speaking hypothetically, they express their concern about engaging with AI and apps as they do not directly “see” a person’s nonverbal response and how they are feeling, therefore missing important information. While this could be considered a general limitation regarding the capability of AI and apps to provide accurate and effective care, this limited capability might be particularly important in high-risk situations, such as during a crisis (as suggested by Meghan). Indeed, research suggests that due to limited verbal communication in a crisis, non-verbal communication may be of particular importance [54]. Thus, while students expressed general concerns about the capability of AI and apps to provide mental health and well-being support, these concerns might be particularly present for crisis situations.

Thus, students were skeptical about whether mental health and well-being can ever be fully comprehended, supported, and promoted by automated digital technologies. Therefore, leading to a lack of trust, which currently prevents them from engaging with AI and apps to support and promote their mental health and well-being.

#### An Inability to Safeguard Mental Health and Well-Being?

Another significant concern students expressed is whether AI and apps can safeguard people’s mental health and well-being. Safeguarding mental health and well-being includes protecting mental health and well-being and preventing harm [55]. AI and apps’ limited capability, and the potential harm that could occur (especially when being used in high-risk situations), raised concerns about the morality and ethicality of using AI for mental health and well-being support. Students would engage with AI and apps for their mental health and well-being more if they were aware of safeguarding systems that are in place and knew that they could trust the apps’ intention and ability to safeguard.

Students identified the importance of mental health and well-being being protected and safeguarded and that this should always be a priority. They mentioned that this should be at the forefront for them to trust AI and apps, and to engage with them for their mental health and well-being. Students identified that AI and apps should only be used for mental health and well-being purposes that are safe and have been proven effective. Furthermore, whenever mental health deteriorates, students identified it is important for AI and apps to recognize this, so the individual can be signposted to health care professionals instead.

*I think that maybe, it should sort of identify a point where, “right this person needs to then be in contact with a person rather than just the app.” … I think knowing the point where you then put them in contact with the person, would be really important.*

[Carry]

Carry's account was considered to reflect a broader pattern across participants, in which human-delivered professional support is constructed as more trustworthy and preferable compared to AI- and app-based alternatives. This is illustrated by Carry's account that safeguarding should be in place by signposting an individual from the AI or app to a mental health care professional when mental health deteriorates. Many students identified that this was the moral and ethical obligation developers have when creating automated digital technologies for mental health and well-being.

*If you were realising that someone was really struggling and then you didn’t offer them [professional support], like in an ethical, moral standpoint, that wouldn’t be very uhm righteous I think, to be almost like “You should stay here and we can solve everything you have.”*

[Colin]

Carry and Colin’s quotes suggest that digital tools should recognize their inabilities and escalate appropriately to human support when users’ mental health worsens to safeguard mental health and well-being. Leo et al [56] similarly found that when mental health symptom severity increased, a preference was displayed for human support compared to digital support—potentially due to individuals’ enhanced trust in the capability and effectiveness of treatment provided by health care professionals.

Nevertheless, students also identified that safeguarding and signposting might be difficult due to the scalability of AI and apps. The great scalability means that many users from different cultures and backgrounds can access the app. This poses risks to safeguarding mental health, as cultural background is an important factor in the etiology of mental health conditions, as well as the expression and diagnosis associated with mental health symptoms [57].

*I think problems could be maybe harder to monitor, because … if so many people are using this app around the world at any moment at any time, it would be very hard to have people … monitoring, uhm, whether there are these sort of safe practises ongoing.*

[Harry]

Harry identifies that it could be challenging to safeguard mental health when many people are using AI and apps worldwide. Safeguarding by ensuring relevant and tailored support is provided to effectively support individuals and prevent harm across different cultural backgrounds is important. Furthermore, as the expression and diagnosis differ across cultural backgrounds, it is important to consider what signposting could be in place to further safeguard mental health and well-being.

### Theme 4: Usage Dependent on Unique AI and App Characteristics

#### Overview

Students identified that AI and apps contain unique characteristics. These unique characteristics impact the situations in which students would engage with them. This is again consistent with the widely used TAM, which suggests that technological characteristics are important in determining engagement [35,58]. Although students primarily referred to AI when discussing specific technological characteristics, they may apply to general apps too.

#### No Judgment or Pressure

AI and apps contain unique characteristics that students found made it a favorable option for promotion and support of mental health and well-being in certain situations. Many students identified a concern about accessing professional support, as this takes up the time of health care workers. Furthermore, students are aware that mental health care services are overwhelmed and that waiting lists are long, thereby feeling guilty about taking up health care workers’ time. In addition, they considered that taking up another person’s time adds a certain “pressure.” Similarly, students felt social support also has a “pressure” associated with it, while automated digital technologies do not contain this “pressure.”

*You don’t feel like you’re sort of wasting a GP’s time.*

[Robert]

*… the pressure’s off ... I feel like [face to face] is more intimidating actually, like talking to someone in person, like expressing your feelings.*

[Sally]

These quotes by Sally and Robert identify the pressure and burden they perceive when accessing professional and social support, taking up another person’s time. Sally identified that she feels more comfortable talking to an AI as there is less “pressure.” Indeed, this was a view reflected by several students.

Another characteristic that students perceive positively is the perceived inability to judge. This was a reason for many students to engage with AI and apps for their mental health and well-being. Many students described being worried or anxious about judgment from others when seeking social support (eg, friends and family) as well as professional support. Talking to an AI or app that is unable to judge them, therefore provides a less intrusive way to access support.

*When I first started therapy, I was not as comfortable expressing everything to my therapist, because at the end of the day, it is a human. You’re always kind of thinking that “oh, what if they judge me though?” [Interviewer: “mm”*] *“What if they think certain things about me” [Interviewer: “mm”]. Therefore applying all that to an app … you can be as blunt and honest as you want [Interviewer: “mm-hmm”].*

[Hayley]

*I think it sort of frees you to be quite open about things. Whereas, you know, in front of someone, you might feel that sort of … judgement of maybe like what you’re saying.*

[Nathalie]

Hayley and Nathalie mention that when using digital technologies, they feel less judged and therefore more comfortable expressing themselves, indicating they feel less disrupted expressing themselves to automated digital technologies compared with people. It has long been established that anxiety and fear of judgment are important factors in determining someone’s willingness to seek help [59] as well as someone’s willingness to be open and discuss certain topics in therapy [60]. Overall, the AI and app characteristic lacking the ability to judge enhances students’ engagement for mental health and well-being purposes.

#### Lack of Humanity, Warmth, and Care

Students further identified that a unique characteristic that is lacking in AI and apps is warmth and humanity, which refers to the kindness and compassion that people have for one another. This kindness and compassion are uniquely shared among humans. Similarly, humans can “care” for one another, which is feeling or showing concern for or kindness to others. According to Watson [61], humans who care are “present, open to compassion, loving, merciful, gentle, loving and kind.” While AI can convincingly imitate some human feelings and expressions, such as kindness or care, through speech, the motivation for these expressions does not stem from innate and underlying feelings of warmth, kindness, and care. Therefore, humanity and warmth are important determinants for the situations in which students may engage in these digital technologies.

*… sometimes you just sort of need a person? … I know there’s a lot that AI and, sort of, apps can do. But I think that, there’s a lot to be said for being able to speak to a person. I think it gives you more of that sort of human element. There’s more humanity in it.*

[Ellie]

Ellie mentions in this quote that AI can “do a lot,” which refers to AI being very capable in creating effective responses. For example, research has found that when individuals are unaware of whether a message is created by AI or a human, AI can at times better detect people’s emotions and show empathy than humans can [62]. Nevertheless, Yin et al [62] also found that when individuals are aware that a response has been generated by AI instead of a human, individuals feel more “heard” by a human despite this response reflecting their emotions less accurately and being less empathetic. People adopting AI in the real world are aware they are talking to AI chatbots, and this could lead them to feel less heard. However, this lack of humanity seems imperative only when an individual needs humanity (eg, when they want someone to care for them, or they want to feel understood).

*It depends on the circumstance and also, uhm, the reason why you decide to go to the app in the first place.*

[Sarah]

Sarah identifies that not every goal or purpose might require humanity. This is in line with recent research findings that AI can effectively deliver a range of different strategies that promote and support mental health and well-being that do not require humanity, such as psychoeducation, treatment adherence, and positive psychology techniques [63]. Thus, humanity, warmth, and care can function as a barrier to engagement in certain situations. However, most individuals identified a range of situations in which AI and apps could be beneficial despite the lack of humanity, warmth, and care.

Overall, AI and apps contain unique characteristics that impact the way and situations in which individuals would engage with them for their mental health and well-being. Specifically, it might provide a less intrusive way to access mental health support due to the lack of pressure. Furthermore, individuals might be more likely to engage in situations in which a lack of judgment may be more imperative, or when they are in need of support that does not require humanity, warmth, and care.

## Discussion

### Summary of the Findings

This study aimed to understand nonclinical students’ experiences of engaging with AI and apps to promote and support their mental health and well-being. A reflexive TA of 21 interviews led to the construction of 4 main themes and 4 subthemes. The main themes identified how (1) experiencing a “need” to engage, (2) stigma as a barrier and solution, (3) a lack of trust, and (4) usage dependent on unique AI and app characteristics can impact students’ engagement with apps and AI for their mental health and well-being. Furthermore, in the theme “lack of trust,” the subthemes “insufficient capability to support and promote mental health and well-being?” and “an inability to safeguard mental health and well-being?” highlight the lack of trust students currently experience in AI and apps to effectively support their mental health and well-being, thereby reducing their engagement. Finally, the theme “usage dependent on unique AI and app characteristics” consisted of subthemes “no judgment and pressure” and “a lack of humanity, warmth, and care,” showing the unique characteristics of AI and apps, which determine the situations in which students are likely to engage with these digital technologies for their mental health and well-being.

### Positioning of the Study Findings

It is important to reflect on the positioning of the findings of this study to consider its transferability. First, this research was conducted at a university in the United Kingdom, situating the findings within a Western cultural context and a highly educated sample. The majority of students at this university were undergraduate, home-based students, although a growing proportion (28,957/103,618, 23%) were international students [64]. At the time of this study, 65% (12,970/19,770) of students at the university identified as White, while 35% (6800/19,770) identified as belonging to Black, Asian, and ethnic minority groups. According to the TAM, such demographic variables influence engagement with digital interventions [58]. Therefore, the positioning of our participants in a Western university context limits transferability of the findings; for example, to other population subgroups with similar demographics (such as university students in a Western context, which includes hundreds of millions of students).

Second, recruitment strategies focused on the departments of Psychology and Computer Science, further positioning the sample as individuals with greater knowledge of mental health and well-being and/or digital technologies. The TAM suggests how a range of factors, such as self-efficacy, perceived ease of use, and technology anxiety, among others, play an important role in determining engagement with digital interventions. The study population’s enhanced knowledge may lead to greater self-efficacy, ease of use, and reduced anxiety of adopting technology and/or mental health and well-being activities. Hence, this limits the transferability of the findings to other population subgroups with greater knowledge of digital and/or mental health and well-being.

Finally, the rapid and ongoing development of GenAI positions this study temporally. Specifically, findings reflect participant perceptions formed during a specific technological and social context during which GenAI was novel while still rapidly developing. The TAM suggests that subjective norms and attitudes are important in determining engagement with digital technologies [58]. The subjective norms and attitudes participants may have held toward AI and apps may be temporally bounded. Nevertheless, the findings of this study hold relevance for similar populations and contexts, and can provide meaningful contributions to relevant theoretical frameworks, conceptual debates, and practical implications, which are explored in the following sections [65].

### Contribution to Knowledge

#### Experiencing a “Need” to Engage

The first constructed theme suggests that a “need” is required for nonclinical students to engage with fully automated digital technologies for their mental health and well-being. Indeed, research has found that mental health “need” is an important predictor of engagement with digital mental well-being interventions [23]. This finding is also in line with the TAM, suggesting that “usefulness” of a digital intervention determines engagement. Nonclinical university students may find a digital well-being intervention less useful when their mental health and well-being are considered good. However, this contradicts Maslow’s hierarchy of needs, suggesting that self-actualization is a growth need [33]. According to this theory, individuals engage in mental well-being activities from a desire to improve upon their mental well-being as opposed to a “need.” In line with Maslow’s hierarchy of needs, this study found that individuals engage in self-actualization through nondigital activities (such as sports, social activities, hobbies, etc) without experiencing a “need,” suggesting self-actualization could be considered a growth need. Thus, perhaps the digital well-being interventions are uniquely perceived as activities that are “useful” to target deficiency needs but not growth needs.

The BPNT could provide an explanation for this. It suggests that individuals might not use fully automated digital interventions for self-actualization as these interventions may not be as beneficial as nondigital interventions. The BPNT suggests that individuals experience greater mental well-being benefits when the need for “relatedness” is met [36]. The need for relatedness may not be met to the same extent by a fully automated digital intervention as compared with nondigital interventions, and therefore, individuals may turn to digital interventions less for self-actualization. Indeed, relevant evidence suggests that fully automated digital well-being interventions are less beneficial compared to nondigital interventions for self-actualization [15]. However, this does not explain why some individuals do access fully automated digital interventions for self-actualization. Research has identified that nonclinical students engage and continue engaging with mental health and well-being apps in the real-world [66]. Therefore, future research should be conducted to understand why some nonclinical students may engage with fully automated digital interventions for the purpose of self-actualization.

#### A Barrier and Solution to Stigma

A second constructed theme suggests stigma is an important factor in determining engagement. This is consistent with much research on help-seeking for mental health and well-being [40,41,43]. It is important to consider that different factors and indicators relate to stigma across different cultures and countries [67]. Hence, these findings are positioned in a specific setting (Bath, United Kingdom), which has likely influenced the findings. For example, research has found that stigma is more prominent in Eastern compared with Western cultures [68]. Therefore, it is important to consider that stigma may have a different impact across cultural contexts (eg, cultures with increased stigma may be more likely to engage in apps compared with in-person support, as they are even more likely to hide accessing support) [68]. Thus, the findings of this study are consistent with relevant research in Western contexts, but the transferability of the findings to other cultural contexts is limited.

#### Lack of Trust

The third constructed theme suggests that a lack of trust in the capability of digital interventions to support mental health and well-being, alongside a lack of trust in the ability of these tools to safeguard mental health and well-being, reduces engagement. Indeed, the TAM suggests that trust can impact upon intentions to engage with digital interventions [58]. Interestingly, the updated versions of TAM largely omit the impact of technological capability on engagement, while the findings of this study might suggest that the capability of digital technology could impact trust, which might in turn lead to greater engagement. Additionally, research findings regarding the importance of trust in determining engagement are conflicting. Some research highlights the importance of “trust” in determining engagement with digital technologies [26], whereas other research does not find this [23]. Therefore, additional research is needed to understand the relationship between trust and engagement in digital mental well-being interventions.

Furthermore, it is important to consider that while this study found that digital tools may inherently be perceived as less capable and trustworthy than humans, apps and AI are becoming increasingly capable, which may (in part) diminish the impact of “trust” in determining engagement with digital mental well-being interventions. For example, they are increasingly able to simulate human-like conversations [69]. This might impact upon the ability of apps to deliver mental health and well-being promotion and support strategies, as research has found that “trust” is increased when systems act more human-like. Therefore, this finding may (in part) be temporally bounded.

#### Unique AI and App Characteristics

The final constructed theme suggests that unique AI and app characteristics, such as “a lack of humanity, warmth, and care,” and “a lack of judgment and pressure,” determine engagement. The TAM indeed suggests that technological characteristics could impact upon the engagement with digital technology, although it does not suggest specific characteristics that may be important to consider. Similarly, previous research suggests that technology-related constructs can impact upon engagement [23,24], but this research does not suggest specific factors such as humanity or lack of judgment as important technological characteristics determining engagement in AI and apps for mental health and well-being purposes. Interestingly, while previous research suggests that a need for social connection is an important facilitator to engage in mental health apps [23,26], this study suggests that a lack of social connection may influence the specific contexts in which individuals engage. Perhaps this was found as this study focuses on fully automated digital interventions only, whereas previous research often focuses on nonautomated digital interventions. Nevertheless, this suggests that the situations in which individuals may engage in fully automated digital interventions compared with nonautomated interventions may differ. Future research is needed to explore this further.

### Limitations of the Findings

This study has several limitations that should be considered when interpreting the findings. One study limitation is that students referred to mental well-being promotion and mental health support interchangeably. Initially, the interview focused on mental well-being promotion entirely, and therefore, a definition of mental well-being promotion was provided at the start of the interview (Multimedia Appendix 1). Despite this, participants frequently drifted from the definition to discuss the relevance of apps for mental health support instead. Participants’ interchangeable use of these terms limits the findings, as it remains unclear whether reported experiences of engaging with apps and AI relate to mental well-being promotion, mental health support, or both. Similarly, participants’ interchangeable reference to apps and AI makes it unclear whether these experiences relate specifically to apps, AI, or both.

Furthermore, while the research question was refined to include mental health support (which is considered good practice when adopting a reflexive qualitative approach [70]), the initial interview schedule did not include questions relating to mental health support. Therefore, findings relating to mental health support and crisis situations were entirely emergent and did not derive from systematic questioning across participants. This limits the representativeness of these findings. Therefore, future research should adopt systematic questioning across participants for more representative insights into their perceptions of engagement with apps for mental health support and during periods of crisis.

Another limitation is that complexities and contradictions may have been lost as the dataset was analyzed cross-cases. This is a particular issue in larger samples [28]. When focusing on patterns, the commonalities in the dataset are identified. Nevertheless, it is important to recognize that individual differences exist. For example, the TAM identifies the importance of demographic and personality characteristics impacting engagement with digital technologies [58]. Similarly, we found that while most students would only use AI and apps for mental health and well-being when in “need,” some students would continually engage to promote their mental well-being. This loss of data is called the “fragmentation of accounts” [71], and while this means there is a loss of contradiction within the dataset, the study’s aim was to provide a general overview of the commonly experienced factors to engage with AI and apps for their mental health and well-being. Nevertheless, it is important to consider that differences in perceptions and engagement exist across individual differences, which should be further investigated in future research.

Finally, a limitation of the study was that no demographic data were collected. While demographic data were not collected in order to protect participants’ confidentiality, this limits the contextual understanding of the findings in this study. For example, it remains unclear whether participants were primarily male or female, and research has shown that sex (among other demographic factors) is important in determining the acceptance and engagement with digital technology [58]. Nevertheless, despite this limitation, the National Institute for Health and Care Excellence suggests that clear reporting of the recruitment strategy (eg, recruitment within the department of Computer Science and Psychology) can still contextualize the study findings to some extent [11].

### Directions for Future Research

The themes constructed in this inductive reflexive TA have identified many different areas for future research into AI and apps to promote and support mental health and well-being in students. First, since students primarily identified interest in engaging in times of “need,” future research could focus on engagement and the effectiveness of adopting these unguided self-help interventions during times of “need.” Indeed, a recent systematic review aimed to understand engagement with unguided self-help apps in clinical populations but found insufficient evidence [72]. Thus, it remains unclear how effective these interventions are when in “need.”

Second, while this study presents common perceptions and experiences regarding engagement, such as most students primarily being interested in engaging for mental health support, it is important to consider that individual differences exist. Indeed, Borghouts et al [23] found that individual differences impacted engagement with digital technologies. However, it remains unclear which individual differences impact engagement with and the effectiveness of digital mental well-being promotion [15]. Thus, future research should investigate the impact of individual differences in engagement with AI and apps for mental health and well-being promotion.

Third, while stigma has been identified as a barrier to engagement, the current findings propose that AI and apps could help reduce public and self-stigma. Either through a stepped-care model [44], or through effective activities combating stigma being incorporated into apps [45]. Nevertheless, future research is needed to investigate whether a stepped-care model that incorporates apps and engagement with activities within apps that reduce stigma could ease the public and self-stigma in a UK university setting.

Finally, this study identified that building trust in digital health technologies is an important determinant of engagement with AI and apps for mental health and well-being support. However, it was also identified that mistrust is occasionally warranted due to the limitations in AI and app capabilities [50]. Future research should therefore establish when and under what conditions AI and apps can be “trusted.” It should establish when and under what conditions AI and apps are safe to use and effective for mental health and well-being support. This future research would be essential to ensure appropriate recommendations regarding engagement can be made and to foster justified trust in these interventions.

### Implications for Policy and Practice

Adopting an inductive thematic approach in this study allowed the generation of unanticipated insights and insights with actionable outcomes that could shape policy development [28]. Several implications for policy and actionable outcomes for AI and app developers are identified.

First, the study found that stigma was perceived as a common barrier to engagement in a UK setting. Therefore, UK policy and practice should consider ways to reduce the stigma associated with AI and apps for mental health and well-being purposes. Ho et al [44] suggest that less intensive support may be considered less stigmatizing. Hence, it may be important for digital mental well-being promotion to be advertised as distinct from more intensive digital mental health support to reduce stigma. Furthermore, other methods could be adopted to reduce public and self-stigma, including adopting education-based programs [73].

Furthermore, developers should consider whether developing AI and apps for the purpose of mental well-being promotion is most beneficial to nonclinical students. The World Health Organization suggests mental well-being promotion can be developed through universal interventions [30] that are aimed at general (nonclinical) population groups who are not specifically “at-risk” due to lower levels of mental health or well-being. Furthermore, developing these interventions digitally provides many benefits. However, this study suggests that mental well-being promotion interventions may be more engaging when delivered nondigitally. It also suggests that students are more willing to engage with AI and apps in times of “need.” Thus, perhaps future AI and app development should primarily focus on supporting mental health conditions instead of promoting mental well-being.

Finally, policy and practice should incorporate ways to safeguard mental health when making AI and apps publicly available. Mental health conditions may present and can be identified differently across cultures [57], making it more challenging to safeguard mental health when these automated digital technologies are publicly available worldwide. This may be particularly challenging, as identifying high-risk individuals may have consequences for data protection and security [74]. Nevertheless, to build trust and enhance engagement with AI and apps, further strategies should be adopted by developers to safeguard mental health and well-being.

### Conclusion

In conclusion, this study aimed to understand university students’ experiences and perspectives of engaging with AI and apps to promote and support their mental health and well-being. Several themes were constructed by the research team, identifying that (1) most students have to experience a “need” to engage; (2) engagement may be impeded by stigma in university students in the UK, although AI and apps might also provide a promising solution to stigma through a stepped-care model and educational activities; (3) a lack of trust in the capability of AI and apps for mental health and well-being and its ability to safeguard are currently perceived as barriers to engagement, and policy and practice should consider ways to build trust to enhance engagement; and (4) unique AI and app characteristics may determine in what situations individuals may engage. Future research should investigate for whom these digital technologies may be engaging in nonclinical populations and when trust in AI and apps for mental health and well-being support may be warranted. Finally, future policy and practice should adopt strategies to reduce public and self-stigma and build trust by incorporating safeguarding strategies for mental health and well-being, particularly when scaling these automated digital technologies worldwide. Nevertheless, these accessible and cost-effective interventions provide a promising method to support mental health and well-being in university students.

## Supplementary material

10.2196/75381 Multimedia Appendix 1 Interview schedule.

10.2196/75381 Multimedia Appendix 2 Information sheet.

10.2196/75381 Multimedia Appendix 3 Consent form.

10.2196/75381 Multimedia Appendix 4 Debrief form.

10.2196/75381 Multimedia Appendix 5 Overview of artificial intelligence (AI) and general apps (these apps may include AI features).

## Abbreviations

AI: artificial intelligence
BPNT: basic psychological needs theory
GenAI: generative artificial intelligence
TA: thematic analysis
TAM: technology acceptance model
