# Supplementary material for: Understanding University Students’ Experiences of Engaging With AI and Apps for Their Mental Health and Well-Being: Qualitative Study
Source: J Med Internet Res. 2026 Jun 30;28:e75381. doi: 10.2196/75381 (PMC13317676; doi:10.2196/75381)
Supplement: Multimedia Appendix 1 [file jmir-v28-e75381-s001.docx]

**Part S1. Semi-structured interview questions**

1. What do you perceive as mental well-being promotion?

*[Provide definition of mental well-being promotion]*

1. What do you perceive as digital mental well-being promotion?
2. What are your thoughts on using an app specifically, to promote your mental well-being?

*Prompts: Can you think of anything positive or negative about mental well-being promotion apps?*

1. Have you used mental well-being promotion apps?

*IF YES, Prompts: What did you like/dislike about them? How have you used them before? (e.g. When? Where? How often?).*

*IF NO, Prompts: What is the reason you haven’t used them before?*

1. Would you currently consider using a mental well-being promotion app?

*Could you tell me a bit more about that? How? When? Where? Why?*

1. What might stop you from using a mental well-being promotion app?

*Prompts: Personal, social, environmental, practical.*

1. What could make you more likely to engage in/use a mental well-being promotion app currently?

*Prompts: Personal, social, environmental, practical.*

**Part S2. Thinkaloud questions**

***Note: This part of the interview has been analysed and presented separately and is not reflected in the current manuscript.***

While looking at the app, please feel free to share your thoughts about the app out loud.

Prompts:

- What are your first thoughts about this information/activity/session?
- What are you thinking about the information now?
- Can you tell me a bit more about why you think that?
- Can you tell me what it is that you like/don’t like about that?
- How do you feel about that now?
- What do you think about that information at the moment?
- Overall, how do you feel about the mental well-being app?
- Can you tell me about anything you felt was good about the content/information?
- Can you tell me anything about the app that you were less keen on or felt was less relevant to you?
- What do you think should be changed?
- Is there anything else you would like to talk about that we haven’t already discussed?
