# Supplementary material for: Understanding University Students’ Experiences of Engaging With AI and Apps for Their Mental Health and Well-Being: Qualitative Study
Source: J Med Internet Res. 2026 Jun 30;28:e75381. doi: 10.2196/75381 (PMC13317676; doi:10.2196/75381)
Supplement: Multimedia Appendix 2 [file jmir-v28-e75381-s002.docx]

**
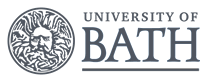
**

INFORMATION SHEET

Understanding students’ perceptions of digital mental well-being promotion.

What is this study about?

This study aims to understand students’ perceptions of digital mental well-being promotion to inform future interventions and research.

The study is being carried out by researchers from the University of Bath.

What will happen if I decide to take part?

You have completed some brief online questionnaires to test that you were eligible to take part. Since you were considered eligible, the researcher contacted you to schedule a convenient date and time for an in-person one-to-one interview in the lab on the University of Bath campus in 10West.

Next, the interview will include a discussion about digital mental well-being promotion and viewing some digital mental well-being promotion app content - you’ll be asked to share your thoughts on this. The interview will be recorded for analysis purposes.

Following the interview, you will be debriefed and reimbursed for your time with a 10-pound Amazon e-gift voucher or university credits, depending on your preference.

How long will the study take?

The interview will take approximately 1 hour.

Am I eligible to take part?

To take part in this study you must:

- Be a student
- Be aged 18+
- NOT currently receive mental health care
- NOT currently experience anxiety or depression

What are the risks and benefits of taking part?

The benefits of taking part in the current study are that you are aiding research into digital mental well-being interventions to support good mental well-being in students and that you will be reimbursed 10 pounds or university credits for your time.

The interview is not expected to cause any harm to you. However, if you do experience any psychological distress during the interview when discussing mental well-being, you can pause or stop the interview at any time by informing the researcher.

Mental health support services are available should you need/want this. You can contact student support services at the university, your GP or the NHS (dialling 111) for mental health support. If you have any minor mental health queries you can visit the MIND website.

How will my data be used?

Personal data such as your name and email address, will only be used for the purpose of the current study. It will be stored securely in an encrypted file on a secure University of Bath server and only members of the research team will be allowed to see your personal data. This data will be destroyed once the study has finished.

The interviews will be recorded and transcribed for analysis purposes. This data will be stored in the same way. After the study finishes, this data will be completely anonymised, so you won’t be identifiable from this data. Anonymised quotes may be shared with the wider research team, and published in reports or papers.

We aim to use the data collected in this study to inform future digital mental well-being promotion strategies to promote mental well-being in the general population more efficiently.

Do I have to take part? And can I withdraw from the study later?

No, you do not have to take part in the study if you don’t want to. Participation in the study is completely voluntary. If you do decide to take part, you can still withdraw from the study at any time. You do not have to give a reason for this. You can withdraw from the study by informing Julia Groot about this verbally during the interview or via email: [jmdg20@bath.ac.uk](mailto:jmdg20@bath.ac.uk) once the interview has finished. You can also request that your data is withdrawn and deleted up to 31/05/2023. After this date, your personal data will have been destroyed and any other data completely anonymised, so we won’t be able to identify and destroy your data anymore.

Who is running the study?

This study has been reviewed and approved by the Psychology Research Ethics Committee (PREC) at the University of Bath (reference number: 23-042), email address: [psychology-ethics@bath.ac.uk](mailto:psychology-ethics@bath.ac.uk)

The project is led by Julia Groot (PhD Researcher) and supervised by Professor Mark Brosnan at the Department of Psychology, Dr Christopher Clarke at the Department of Computer Sciences, University of Bath, and Dr Ben Ainsworth at the School of Psychology, University of Southampton. Julia Groot is leading the day to day running of the project as part of her PhD research. If you would like to discuss any aspect of the research, you can ask or email Julia Groot on [jmdg20@bath.ac.uk](mailto:jmdg20@bath.ac.uk).
