# Supplementary material for: Understanding University Students’ Experiences of Engaging With AI and Apps for Their Mental Health and Well-Being: Qualitative Study
Source: J Med Internet Res. 2026 Jun 30;28:e75381. doi: 10.2196/75381 (PMC13317676; doi:10.2196/75381)
Supplement: Multimedia Appendix 3 [file jmir-v28-e75381-s003.docx]

**
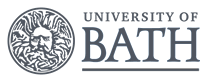
**

**CONSENT FORM**

**Understanding students’ perspectives on digital mental well-being promotion.**

**Please answer the following questions to the best of your knowledge**

**YES NO**

**Do You confirM That you:**

- are a student? **□ □**
- are aged 18+?  **□ □**
- do NOT currently receive mental health care (treatment or psychopharmaceutical drugs) **□ □**
- do NOT currently experience anxiety or depression? **□ □**

**Have you:**

- been given information explaining about the study? **□ □**
- had an opportunity to ask questions and discuss this study? **□ □**
- received satisfactory answers to all questions you asked? **□ □**
- received enough information about the study for you to make a decision

about your participation? **□ □**

**Do you understand:**

that you are free to withdraw from the study and free to withdraw your data prior to anonymisation

- at any time? **□ □**
- without having to give a reason for withdrawing? **□ □**

**I hereby fully and freely consent to my participation in this study**

I understand the nature and purpose of the procedures involved in this study. These have been communicated to me on the information sheet accompanying this form.

I understand and acknowledge that the investigation is designed to promote scientific knowledge and that the University of Bath will use the data I provide for no purpose other than research.

I understand that the data I provide will be kept **confidential**, and that on completion of the study my data will be **anonymised** by removing all links between my name or other identifying information and my study data. This will be done by 31/05/2023, and before any presentation or publication of my data.

I understand that after the study will be made “open data”. I understand that this means the anonymised data will be publicly available and may be used for purposes not related to this study, and it will not be possible to identify me from these data.

Participant’s signature: ________________________________ Date: ________________

Name in BLOCK Letters: _____________________________________

**Final consent**

**Having participated in this study**

I agree to the University of Bath keeping and processing the data I have provided during the course of this study in accordance with the information I received at the outset and the Data Protection Regulation.

Participant’s signature: _________________________________ Date: ________________

Name in BLOCK Letters: _____________________________________

If you have any concerns related to your participation in this study please contact the Department of Psychology Research Ethics Committee, via email: psychology-ethics@bath.ac.uk.
