# Supplementary material for: Understanding University Students’ Experiences of Engaging With AI and Apps for Their Mental Health and Well-Being: Qualitative Study
Source: J Med Internet Res. 2026 Jun 30;28:e75381. doi: 10.2196/75381 (PMC13317676; doi:10.2196/75381)
Supplement: Multimedia Appendix 4 [file jmir-v28-e75381-s004.docx]

**
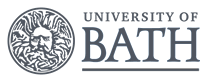
**

**DEBRIEF**

**Understanding students’ perspectives of digital mental well-being promotion.**

**Background and aims of the study**

Promoting mental well-being in the general population is important; it allows people to feel more positive and teaches essential skills and knowledge which can protect people from developing a mental illness. However, few people continue using digital mental well-being apps, despite their great potential for improving mental well-being in the general population. Therefore, the study aimed to understand perspectives about digital mental well-being promotion to identify any potential barriers and facilitators to using a mental well-being promotion app.

This understanding allows us to make better recommendations on how to adapt digital mental well-being promotion apps to be more engaging and effectively promote mental well-being.

**What happens next?**

We will transcribe the recording to ensure we are accurately recording your views. We will ensure that any identifiable information in the transcripts is removed to ensure confidentiality. The data will then be analysed. We will then write-up the results which will inform the ongoing development of a mental well-being promotion app and future research. If you’re interested to learn more about the study results, feel free to contact the main study lead about this (see contact details below). We also aim to make the results available by publishing them online in a relevant journal.

Please be aware that you can still contact the study lead until 31/05/2023 if you would like to withdraw your data (see contact details below). After this, withdrawal from the study is no longer possible. This is because your personal data will be destroyed by then and all the data will be fully anonymous, so we will no longer be able to identify and destroy your data.

**Useful readings**

If you would like to read more about digital mental well-being promotion, see:

What works centre: <https://whatworkswellbeing.org/about-wellbeing/>

Positive psychology: <https://positivepsychology.com/positive-psychology-interventions/#interventions>

**Further Support**

Further support and advice on how to engage in activities to promote your mental health and well-being can be found here:

Student support: <https://www.bath.ac.uk/professional-services/therapeutic-services-mental-health/>

MIND for further information and support on mental health: <https://www.mind.org.uk/>

NHS 5-steps to mental well-being: <https://www.nhs.uk/mental-health/self-help/guides-tools-and-activities/five-steps-to-mental-wellbeing/> ]

**Contact Details**

If you have any other questions or would like more information about the study, please feel free to contact the study lead:

Name: Julia Groot

Email address: [jmdg20@bath.ac.uk](mailto:jmdg20@bath.ac.uk)
