# Supplementary material for: Understanding University Students’ Experiences of Engaging With AI and Apps for Their Mental Health and Well-Being: Qualitative Study
Source: J Med Internet Res. 2026 Jun 30;28:e75381. doi: 10.2196/75381 (PMC13317676; doi:10.2196/75381)
Supplement: Multimedia Appendix 5 [file jmir-v28-e75381-s005.docx]

This is an overview of the apps and AI referred to during interviews which were identified through inductive coding.

General apps:

- Habit tracker app
- Mood tracker app
- Journaling, gratitude and reflections app
- Mindfulness/breathing exercises app
- Well-being app by the University
- Generic app with chat function
- Online therapist app
- Chatbot app
- Exercise app
- Sleep tracker app
- Distraction / games app
- Online therapist app
- Screen time app
- CBT app (such as recording thoughts/thought patterns)
- Mental health support chatbot
- Yoga app
- Apps which incorporate multiple well-being strategies
- App for depression
- App for anxiety
- Meditation app
- Cognitive training app

AI-based systems:

- AI therapist app
- App which contains AI to tailor/personalise content
- Websites/apps using AI (such as chatbots)
- General LLMs (such as ChatGPT)
- App which incorporates AI

*Note: Inductive coding misses any references to apps/AI which may not have been made explicit by the participant.*
